# Temporal Regulation of* fim* Genes in Uropathogenic* Escherichia coli* during Infection of the Murine Urinary Tract

**DOI:** 10.1155/2017/8694356

**Published:** 2017-12-27

**Authors:** William R. Schwan, Hua Ding

**Affiliations:** University of Wisconsin-La Crosse, La Crosse, WI, USA

## Abstract

Uropathogenic* Escherichia coli* (UPEC) adhere to cells in the human urinary tract via type 1 pili that undergo phase variation where a 314-bp* fimS* DNA element flips between Phase-ON and Phase-OFF orientations through two site-specific recombinases, FimB and FimE. Three* fim-lux* operon transcriptional fusions were created and moved into the clinical UPEC isolate NU149 to determine their temporal regulation in UPEC growing in the urinary tract. Within murine urinary tracts, the UPEC strains demonstrated elevated transcription of* fimA* and* fimB* early in the infection, but lower transcription by the fifth day in murine kidneys. In contrast,* fimE* transcription was much lower than either* fimA* or* fimB* early, increased markedly at 24 h after inoculation, and then dropped five days after inoculation. Positioning of* fimS* was primarily in the Phase-ON position over the time span in UPEC infected bladders, whereas in UPEC infected murine kidneys the Phase-OFF orientation was favored by the fifth day after inoculation. Hemagglutination titers with guinea pig erythrocytes remained constant in UPEC growing in infected murine bladders but fell substantially in UPEC infected kidneys over time. Our results show temporal* in vivo* regulation of* fim* gene expression in different environmental niches when UPEC infects the murine urinary tract.

## 1. Introduction

Urinary tract infections (UTIs) remain one of the most common infections of humans in the United States. Approximately 10.5 million office visits are due to UTIs annually, resulting in over 100,000 hospitalizations and an estimated cost of $3.5 billion per year [1–3]. More than 80% of all UTIs are due to uropathogenic* Escherichia coli* (UPEC), causing substantial morbidity and mortality, particularly from the risk of sepsis during pyelonephritis [2].

The ability to bind to uroepithelial cells lining the human urinary tract is generally considered one of the first steps in UPEC initiated UTIs. Type 1 pili facilitate this binding to epithelial cells in the bladder, lungs, intestine, and buccal cells; proximal tubular cells of the kidney; and various inflammatory cells [4–6]. Following adherence of the UPEC cells, bacterial invasion and persistence in target host cells due to the type 1 pili expressed by UPEC can occur [4, 7, 8].

Expression of FimA, the main structural subunit of the type 1 pili encoded by the* fimA* gene [9, 10], is affected by phase variation, a ON-OFF switching process that allows individual cells to alternate between piliated (Phase-ON) and non-type 1 piliated states (Phase-OFF) [11, 12]. This phase switching is due to the inversion of a 314-bp* fimS* DNA element containing the promoter for the* fimA* structural gene [13, 14]. When the* fimA *promoter is aligned in the Phase-ON orientation, transcription of* fimA *occurs. However, when the* fimS *element is in the Phase-OFF orientation, there is no transcription of* fimA*, resulting in a non-type 1 piliated phenotype [15, 16]. The phase switching of the 314-bp* fimS *sequence is controlled by the products of two regulatory genes,* fimB* and* fimE*, located upstream of* fimA* [16]. The* fimB *and* fimE* gene products are site-specific recombinases influence the positioning of the* fimS* region [16–18]. FimE appears to promote inversion of the promoter-containing* fimS* element from the Phase-ON to Phase-OFF orientation [18, 19], whereas FimB promotes switching in both directions with a slight switching bias toward the Phase-ON orientation [16, 18, 20].

Both of the* fim* recombinase genes are transcribed independently. The consensus is that there are two* fimB* promoters [21–23], although one study with an* E. coli* K-12 strain has indicated a single promoter for* fimB* [24]. A third potential* fimB* promoter was also identified in UPEC strains [23] that may be tied to sialic acid concentration in the urinary tract [25], but this has not been confirmed. A single promoter has been identified for* fimE* [24]. Regulation of the* fimB* and* fimE* genes in UPEC cells growing in the human urinary tract and other mammals is still largely uncharacterized.

Inside the urinary tract, UPEC grow in an environment bathed in urine. Human and murine urine typically have a slightly acidic pH and the osmolality can vary [26, 27]. Previous work in our laboratory has demonstrated that pH and osmotic changes in growth media have an effect on* fim* gene expression [28, 29]. Transcription of* fimA*,* fimB*, and* fimE* were reduced in the bacteria growing in acidic Luria broth (LB) medium. Previously, it was shown that growth of* E. coli* in medium with a combination of an acidic pH and high osmolality resulted in a significant decline in* fimB* and* fimA* transcription compared to growth in neutral pH/low osmolality medium [29].

Although there have been studies that have examined* fim* gene expression in UPEC colonizing a murine urinary tract, only a limited number of studies had examined the expression of type 1 pili in UPEC growing* in vivo* [30–33]. More studies have examined positioning of the* fimS* element in UPEC strains infected murine bladders and kidneys [34–40]. A few studies have examined the expression of* fimA* in UPEC infecting murine bladders [30, 35]. However, only one study has examined* fimB* expression in UPEC growing in murine bladders, but this study was limited to a 48 h period and did not examine* fim* recombinase gene transcription in infected murine kidneys [35].

In order to address whether there is temporal regulation of* fim* genes in UPEC cells growing in murine urinary tracts, we constructed* fimA, fimB, and fimE-lux* transcriptional fusions and moved these fusions into a UPEC strain. We used these recombinant UPEC strains to infect murine urinary tracts and then examined the expression of the* fim* genes over a five-day period. In this study, we have demonstrated that the* fimA*,* fimB*, and* fimE* genes were differentially regulated in* E. coli *colonizing the bladder versus the kidney. Our results may help us understand the temporal regulation of these adhesion genes in* E. coli* colonizing the human urinary tract.

## 2. Materials and Methods

### 2.1. Bacterial Strains, Plasmids, and Growth Conditions

All strains and plasmids used in this study are listed in the Table 1.* E. coli* DH5*α* MCR (Gibco/BBL) was used for all of the cloning and vector construction.* E. coli *strain NU149 is a clinical isolate obtained from a patient with cystitis that expresses type 1 pili, but not P pili [30]. The NU149 strain has been used for* fim *gene transcriptional analyses and type 1 pili expression [24, 28, 31, 41]. All strains were grown statically in Luria-Bertani (LB) broth at 37°C or were passaged on Luria agar (LA) plates incubated at 37°C. For recombinant* E. coli* strains, the following antibiotic concentrations were used unless otherwise noted: ampicillin, 100 *μ*g/ml; chloramphenicol, 12.5 *μ*g/ml; kanamycin, 40 *μ*g/ml; and erythromycin, 150 *μ*g/ml.

### 2.2. Construction of the* fim-lux* Reporter Fusions

To create the* fimA-lux* reporter fusion on the single copy plasmid pPP2-6, pAON-1 plasmid DNA [28] was extracted using a commercial kit (Qiagen). The pAON-1 DNA containing the promoter for* fimA* was digested with the restriction endonuclease enzymes* Eco*RI and* Bam*HI to separate the* fimA* promoter sequence from the pUJ8 backbone [42] and ligated to pHSS22 plasmid DNA [28] cut with the same restriction endonuclease enzymes described above. A transformation into* E. coli* DH5*α* MCR cells was followed by selection on LA plates containing 40 *μ*g kanamycin/ml. One clone, labeled pHD-01, with the proper* Eco*RI and* Bam*HI restriction endonucleases digestion pattern was chosen for additional processing.

Next, pXen5 [43] plasmid DNA was extracted as described above, cut with* Bam*HI, and ligated to* Bam*HI-cut pHD-01 plasmid DNA created above. The ligation DNA was transformed into* E. coli* strain DH5*α* MCR. Plasmid pXen5 contains an erythromycin resistance gene and a promoterless* lux* operon. The transformants were then plated onto LA containing 40 *μ*g kanamycin/ml incubated at 37°C. All transformants were patched onto LA plates containing 150 *μ*g erythromycin/ml as well as LA containing 40 *μ*g kanamycin/ml. Transformants that were kanamycin-resistant (Kan^R^) but erythromycin-sensitive (Erm^S^) were monitored for luminescence above background levels and had plasmid DNA extracted as described above. Aliquots of plasmid DNA from several transformants were then digested with the restriction endonuclease to confirm the insertion of the* lux* operon into pHD-01 plasmid. One clone that showed bioluminescence as well as the proper* Pst*I digestion pattern was named pHD-02 and was chosen for further analysis.

To avoid problems suffered by multicopy-plasmid-based systems, a single copy plasmid pPP2-6 was used as the final vector for the* fimA*-*lux* reporter fusion [28]. The pPP2-6 plasmid has a chloramphenicol resistance gene and an origin of replication to replicate a single copy. Plasmid DNA from pPP2-6 and pHD-02 constructs were digested with the* Not*I restriction endonuclease, ligated together, and transformed into* E. coli* DH5*α* MCR cells. Transformants were plated onto LA containing 12.5 *μ*g chloramphenicol/ml and incubated at 37°C and then screened for bioluminescence as previously described. One transformant that displayed bioluminescence above background levels with the proper* Not*I digestion pattern named pHD-03 was identified. Plasmid pHD-03 was electroporated into electrocompetent strain NU149 cells by a procedure described by Casali and Preston [44], selecting for transformants with 12.5 *μ*g/ml of chloramphenicol. Transformants were screened for bioluminescence. One NU149/pHD-03 clone was chosen for further analysis.

For construction of the* fimE-lux* reporter fusion on the pPP2-6, plasmid pMP5-2.17 containing the* fimE* promoter was used [28]. The pMP5-2.17 plasmid DNA was processed as described above and one clone, labeled pHD-04, was used for further analysis. The* fimE* promoter DNA from pHD-04 was ligated to pXen-5, resulting in the plasmid named pHD-05. Plasmid DNA from pHD-05 was ligated into the pPP2-6 plasmid that resulted in the pHD-06 plasmid.

Construction of the* fimB-lux* reporter fusion was done as follows. The pP5-48 plasmid DNA containing the* fimB* promoters [28] was extracted as described above, leading to creation of the pWS141-2, pWS144-27, and ultimately the pWS145-38 plasmid.

### 2.3. Construction of the* ftsZ-lux* Reporter Fusion

For the housekeeping gene control used in this study, the* ftsZ* gene was chosen, which we have used previously in other studies [28, 31, 45]. To construct the* ftsZ-lux* reporter fusion, the* ftsZ* promoter region was amplified using the primer pair EcFtsZ5 (5′-CAGGAATTCAAACATCGTCAAAGCGGTTGA-3′) and EcFtsZ6 (5′-CAAGGATCCAATTCAACACCTTCAATGCGC-3′) using DNA sequence obtained from one* E. coli* genome sequencing project [46] under the following PCR conditions: initial denaturation at 95°C for 5 min; 35 cycles consisting of denaturation at 95°C for 1 min, annealing at 55°C for 1 min, and elongation at 72°C for 1 min. The final* fts*Z PCR product had an* Eco*RI restriction endonuclease site at the 5′ end and a* Bam*HI restriction endonuclease site at the 3′ end. This* ftsZ* DNA was cut with* Eco*RI and* Bam*HI, ligated to* Eco*RI and* Bam*HI-cut pHSS22 DNA, and transformed into DH5*α* MCR cells. Transformants were selected on LA with kanamycin as described above. One of the resulting transformants was named pHD-07. Plasmid pHD-07 DNA was extracted, cut with* Bam*HI, and ligated to* Bam*HI digested pXen5 DNA, and the ligation mixture was transformed into DH5*α* MCR cells. Transformants were selected on LA with kanamycin and erythromycin and screened for bioluminescence as previously noted. One plasmid, labeled pHD-08, was created. The pHD-05 plasmid DNA was ligated to pPP2-6 DNA and transformed into DH5*α* MCR cells, and selection and screening for bioluminescence were done as described above. One of the plasmids, labeled pHD-09, was transformed into* E. coli* NU149 as described above.

### 2.4. Testing the* fim-lux* Fusions in Different* In Vitro* pH Environments

A previous study has shown that acidic pH and high osmolarity environmental cues regulate the expression of type 1 pili* in vitro* [28] (Schwan et al., 2002). To measure changes following growth in media with different pH and/or osmolarity, LB was buffered using 0.1 M Na_2_HPO_4_-NaH_2_PO_4_ buffer and 1% (vol/vol) glycerol as previously described [28]. The media were separated into a pH ranging between 5.0 and 8.0 with 0.5 pH unit increments. Cultures of* E. coli* NU149 containing* fimA-, fimB-, or fimE-lux* fusions on single copy recombinant plasmids were incubated overnight at 37°C statically in the buffered LB medium at a specific pH. The next day 100 *μ*l of each overnight culture was transferred to another three ml aliquot of buffered LB medium at a specific pH and incubated statically at 37°C until midlogarithmic phase had been reached. Bioluminescence testing was performed as described below.

### 2.5. *In Vitro* Bioluminescence Assays

Each culture was incubated at 37°C statically to midlogarithmic phase. A 500 *μ*l aliquot of each culture was tested for bioluminescence using a FB 12 bioluminescence single tube luminometer (Zylux Corporation). The luminescence results were reported as relative luminescence units (RLU) as described previously [47]. A viable count of each culture was calculated by plating aliquots of 10-fold serially diluted bacteria in phosphate-buffered saline (PBS, [48]) onto LA containing 12.5 *μ*g/ml of chloramphenicol and counting the colonies. The RLU values were divided by the viable counts to achieve RLU/CFU for each culture.

### 2.6. Murine Urinary Tract Infection Model

A murine urinary tract infection model [30] was used to assess the* in vivo* regulation of the* fim-lux* reporter fusions in* E. coli* NU149. The Institutional Animal Care and Use Committee at the University of Wisconsin-La Crosse approved the study design and the animal handling protocols of this study, including the use of isoflurane to anesthetize the mice. All steps were taken to minimize animal suffering throughout the experiment. Briefly, each* E. coli* NU149 strain with a specific* fim-lux *promoter reporter plasmid fusion was grown in LB medium at 37°C statically overnight. One ml culture aliquots were pelleted by centrifugation at 6000 ×g for two minutes and the supernatant was decanted off. Each pellet was suspended in 100 *μ*l of PBS. A 250 *μ*l volume of 10^9^ CFU/ml bacteria was instilled into the urinary bladders of six to twelve female 4- to 6-week-old Swiss Webster mice per time point through a soft polyethylene catheter adapted to a needle. The number of mice represents the aggregate number from at least two batches of inoculations administered on separate days. A larger inoculum volume was used to achieve 80% colonization of the kidneys at most time points and sufficient bioluminescence at the early time points. After 8, 24, 72, and 120 hours after inoculation (hpi), urine was collected to measure the pH and osmolality, and the mice were then euthanized and the bladders and kidneys were removed. Each organ was homogenized in sterile tissue grinders (Kontes) with 1 ml of PBS. The homogenized tissues were tested for bioluminescence as described above. Tenfold serial dilutions of each organ homogenate in PBS for each construct were performed, and aliquots of each dilution were plated in duplicate onto LA containing 12.5 *μ*g chloramphenicol/ml. The plates were incubated overnight at 37°C and the number of colonies per organ homogenate per construct was calculated. The background fluorescence for each organ homogenate was subtracted from the RLU values. Background corrected RLU values were then divided by the number of bacteria determined by the viable bacteria counts, generating corrected RLU/CFU per time point as reported previously [47].

### 2.7. Measurement of Murine Urine pH and Osmolality

Murine urine was collected and the pH measured with pH strips and the osmolality measured using a Reichert TS 400 total solids refractometer (Reichert Analytical Instruments, Buffalo, NY) to assess the specific gravity. The specific gravity readings were converted to osmolality using a chart [29].

### 2.8. PCR for* fimS* Orientation Determination

To determine the orientation of the* fimS* invertible element, previously described PCR techniques were used and products visualized with FOTO/Analyst PC Image Software [41, 45]. To quantify the percentage of Phase-ON or Phase-OFF bacteria, a standard curve was prepared as described by Teng et al. [49] using locked-ON (DH5*α*/pAON-1 [41]) and locked-OFF bacteria (NU149 cells passaged five times on agar shown to be 100* *% Phase-OFF [41]) as PCR templates and the ImageQuant 5.2 software.

### 2.9. Hemagglutination Assays

The HA assays were performed with 1* *% guinea pig erythrocytes (Hardy Diagnostics) as previously described [50], standardizing the HA titer to the viable count. The titers represent the geometric means of ten bladder and ten kidney homogenate samples.

### 2.10. Statistical Analysis

Student's *t*-test was used for statistical analysis of the* in vitro* growth conditions. An ANOVA analysis with a Bonferroni correction was used for* in vivo* analysis from the murine urinary tract organ homogenates. *P* values ≤ 0.05 were considered significant.

## 3. Results

### 3.1. Evaluation of the* fim-lux* Fusions in UPEC Growing in Different pH Media

Previously,* fim-lacZYA* fusions were created and tested in* E. coli* strains growing under* in vitro* conditions [28]. Because of the limitations of using* lacZYA* reporter fusions in UPEC growing in animal tissues, several* fim-lux* transcriptional fusions were created. All the* fim-lux* fusions were created on the single copy number plasmid pPP2-6 and transformed into the clinical* E. coli* strain NU149. Once the* fim-lux* reporter fusions on pPP2-6 were electroporated into strain NU149, each strain containing a* fim-lux* reporter fusion on a single copy plasmid was inoculated into LB adjusted to various pHs that ranged from 5.5 to 8.0 to verify the regulatory patterns that were observed using* lacZ* reporter fusions. The lowest level of sensitivity of these* fim-lux* fusions was from 1.0 to 3.0 × 10^3^ bacterial cells, depending on the fusion tested.

**Figure 1:**
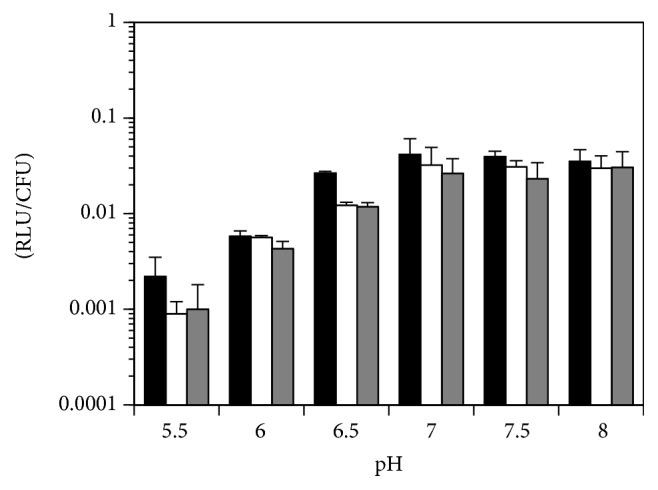
Effects of pH on* fimA *(black column),* fimB *(white column), and* fimE* (gray column) expression as determined with* luxABCDE* transcriptional fusions in strain NU149. RLU/CFU were measured with a luminometer and then divided by viable counts; means ± standard deviations are indicated from at least three separate runs.

When the* E. coli* cells were grown to midlog phase in various pH media, all three of the* fim-lux* fusions (*fimA-lux, fimB*-*lux*, and* fimE-lux*) displayed the lowest level of expression at pH 5.5 (*fimA-lux*, 0.0022 RLU;* fimB-lux*, 0.0009 RLU; and* fimE-lux*, 0.001 RLU) (Figure 1). A shift from pH 5.5 to a neutral pH 7.0 in LB media resulted in dramatically increased expression for all three* fim* genes (*fimA-lux*, 19-fold, *P* < 0.000005;* fimB-lux*, 36-fold, *P* < 0.006; and* fimE-lux*, 26-fold, *P* < 0.00002). When the pH was greater than 7.0, expression of the* fimE-lux* fusion reached the highest level at pH 8.0, whereas* fimA-lux* and* fimB-lux* transcription dropped slightly compared to growth in pH 7.0 LB media. These results confirmed the* fim-lacZ* fusions results that showed that, in a low pH environment, transcription of all of the* fim* genes was repressed.

### 3.2. Temporal Regulation of Transcription from Three* fim* Genes in a UPEC Strain Infecting Murine Bladders versus Kidneys

**Figure 2:**
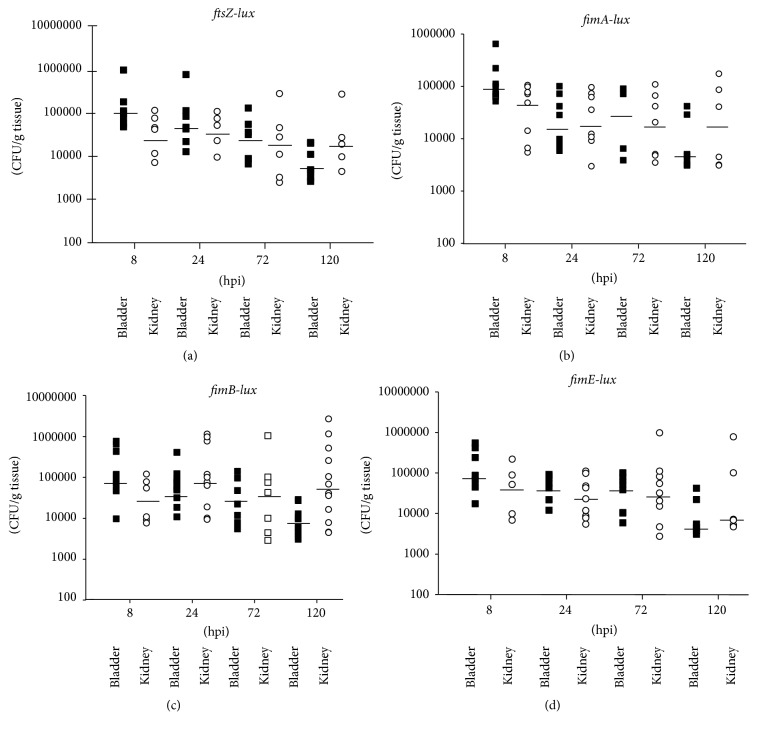
Viable counts of* E. coli* strain NU149 containing different* fim-lux* fusions in infected murine bladders and kidneys after 8, 24, 72, and 120 hours after inoculation (hpi). Murine tissues included bladders (black square) or kidneys (white circle) from the* ftsZ* (a),* fimA* (b),* fimB* (c), and* fimE* (d) fusion cultures. Five to twelve animals per time point were examined where each symbol represents one mouse. The black horizontal bars represent the median values for each time point and tissue.

The* in vitro* analysis described above and our previous study [28] demonstrated that* fim* gene transcription was maximally repressed in the UPEC strains growing in a low pH/high osmolality environment that would mimic the environment found in regions of the murine kidneys. Our next step was to assess expression of the* fim-lux* fusions in the NU149 UPEC strain infecting murine urinary tracts over a five-day period after inoculation. Strains of NU149 with the pHD-03* (fimA-lux)*, pWS145-38* (fimB-lux)*, pHD-06* (fimE-lux)*, and pHD-09* (ftsZ-lux)* plasmids were intraurethrally injected into the murine urinary tracts of female Swiss Webster mice. After 8, 24, 72, and 120 hpi; bladders and kidneys were collected, homogenized, tested for their RLU, and plated for viable bacterial counts to obtain the corrected RLU. Murine urine was also collected and the pH of the murine urine samples ranged from 5.5 to 6.5 with an average pH of 6.0. The osmolality of the murine urine ranged from 450 mOsm to 720 mOsm. Bacterial viable counts in the murine bladders had median values of 7.45 to 9.80 × 10^4^ at 8 hpi, 1.90 to 5.90 × 10^4^ after 24 hpi, 2.24 to 5.20 × 10^4^ at 72 hpi, and 4.8 to 7.9 × 10^3^ after 120 hpi (Figure 2). The median viable counts in the murine kidneys ranged from 3.30 to 6.05 × 10^4^ at 8 hpi, 2.32 to 8.95 × 10^4^ after 24 hpi, 2.08 to 4.30 × 10^4^ at 72 hpi, and 7.05 × 10^3^ to 5.50 × 10^4^ after 120 hpi (Figure 2).

**Figure 3:**
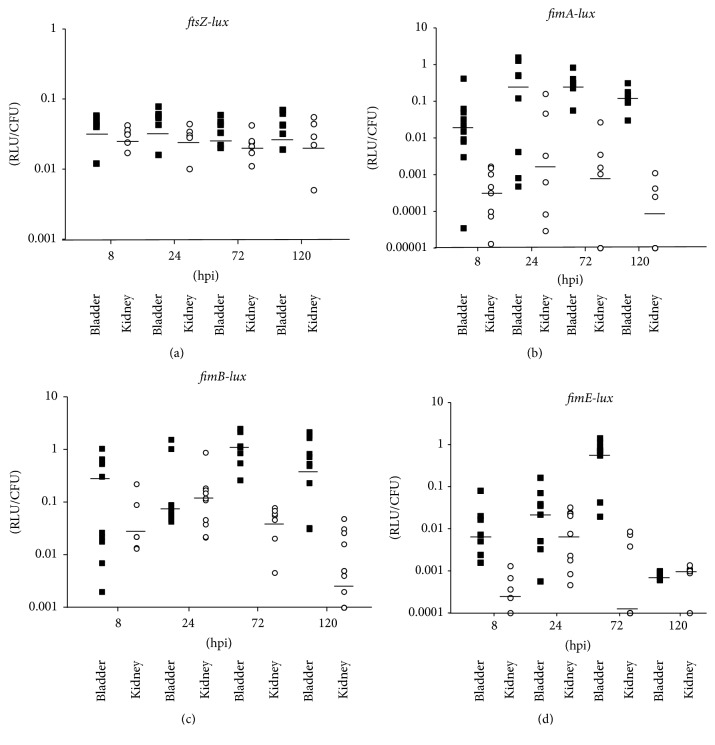
Expression of* ftsZ* (a),* fimA *(b),* fimB *(c), and* fimE *(d) in murine tissues after infection with* E. coli* strain NU149 as determined with* fim::luxABCDE* transcriptional fusions after 8, 24, 72, and 120 hours after inoculation (hpi). Murine tissues included bladder (black square) or kidney (white circle). RLU/CFU were measured with a luminometer, background fluorescence subtracted, and then divided by viable counts; median values are indicated. Five to twelve animals per time point were examined where each symbol represents one mouse. The black horizontal bars represent the median values for each time point and tissue.

For the* ftsZ-lux* fusion, transcription of* ftsZ* in UPEC infected murine bladders showed minimal variation from 0.038 to 0.056 (Figure 3(a)) that was not significant. Compared to* ftsZ* expression in infected bladders,* ftsZ* transcription in UPEC infected murine kidneys showed less fluctuation among the five-day period after inoculation, ranging from 0.023 to 0.032. These results indicated transcription of* ftsZ-lux* fusion was relatively stable with less than 0.1-fold fluctuations in the NU149 infected murine bladders or kidneys.

The* fimA-lux* fusion expression in NU149 infected bladders was at the lowest level at 8 hpi (0.0185 RLU/CFU) and then increased to the highest level (0.305 RLU/CFU) after 24 hpi (Figure 3(b)). Subsequently,* fimA* transcription fell to 0.313 RLU/CFU and 0.122 RLU/CFU at 72 and 120 hpi, respectively. The difference in* fimA* transcription in NU149 infected bladders was not significant (*P* < 0.083). In contrast,* fimA *transcription in NU149 infected murine kidneys increased initially during the first 72 hpi (8 hpi, 0.0004 RLU; 24 hpi, 0.0020 RLU/CFU; and 72 hpi, 0.001 RLU/CFU) but dropped significantly by 120 hpi (0.0001 RLU). At 120 hpi,* fimA* transcription was barely detectable. However, the variation of* fimA* expression in NU149 infected kidneys was not significant during the 120 hpi period (*P* < 0.104). Although these results did not show significant variation in* fimA* transcription over time, the results did show much higher* fimA* expression in the UPEC infected bladders than kidneys.

Transcription of* fimB* in NU149 infected bladders showed a trend similar to the* fimA* expression results. At 8 hpi,* fimB* expression was 0.419 RLU/CFU (Figure 3(c)). After 24 hpi, the RLU/CFU decreased to 0.065 and reached the highest level at 72 hpi (1.113 RLU/CFU). This increase in* fimB* transcription was significant compared to the 8 hpi (2.7-fold, *P* < 0.002). By 120 hpi,* fimB* expression slightly dropped to 0.630 RLU. While* fimB* transcription varied in NU149-infected murine bladders, transcription of* fimB *in NU149 infected murine kidneys increased slightly from 0.022 RLU/CFU at 8 hpi to 0.114 RLU/CFU at 24 hpi, then fell to 0.056 RLU/CFU at 72 hpi, and then dropped again to 0.005 RLU/CFU at 120 hpi. By comparing the* fimB* transcription level at 8 hpi to the 120 hpi results,* fimB* transcription in NU149 infected murine kidneys decreased 4.9-fold (*P* < 0.049). Thus,* fimB* transcription was favored in UPEC infected murine bladders over kidneys.

Unlike the* fimA* and* fimB* transcription results in UPEC infected murine bladders,* fimE* transcription was much lower than either* fimA* or* fimB* transcription. At 8 hpi,* fimE* expression was 0.0072 RLU/CFU (Figure 3(d)). Transcription of* fimE* rose to 0.0284 RLU/CFU after 24 hpi and then reached the highest level (0.72 RLU/CFU) at 72 hpi before falling to the lowest level (0.00088 RLU/CFU) at 120 hpi (*P* < 0.0001) compared to the five-day period after inoculation. In UPEC infected murine kidneys,* fimE* transcription significantly increased 30-fold from 8 hpi (0.00036 RLU) compared to 24 hpi (0.0076, *P* < 0.02). At 72 hpi,* fimE* transcription significantly declined (0.0001 RLU/CFU, *P* < 0.04) and remained down after 120 hpi (0.001 RLU/CFU, *P* < 0.02) compared to the 24 hpi result. By comparing the* fimE* transcription level at 8 hpi to the 120 hpi results,* fimE* transcription in NU149 infected murine kidneys increased 1.8-fold. These results suggested* fimE* transcription first increased and then was repressed in both NU149 infected murine bladders and kidneys, but the final level of* fimE* transcription in NU149 infected murine kidneys increased slightly over the first time point. Thus, the increase in* fimE* transcription combined with the decrease in* fimB* transcription suggested the Phase-OFF orientation driven by FimE recombinase activity might be favored over the five-day infection period in the murine kidneys.

### 3.3. Positioning of the* fimS* Invertible Element Favors a Phase-OFF Orientation over a Five-Day Period in UPEC Infecting Murine Kidneys

The* fim-lux* fusion results demonstrated a temporal regulation of* fimA, fimB*, and* fimE* within a UPEC strain growing in murine urinary tracts. We could not directly compare* fimB* to* fimE* transcript ratios because of the way each* fim-lux* fusion was created as well as there being potential posttranscriptional modification differences. Within the infected murine bladders, our results suggest but do not confirm the ratio of* fimB/fimE* transcripts may favor* fimB* transcription. Presumably, more FimB would mean more Phase-ON orientation for the* fimS* element. However, in murine kidneys, the decline in* fimB *transcripts combined with an increase in* fimE* transcripts imply that more Phase-OFF oriented* fimS *occurs over the five-day period in murine kidneys.

**Figure 4:**
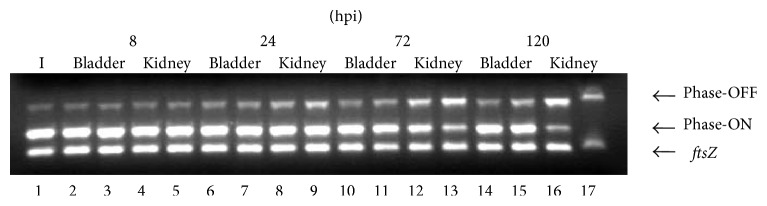
Determination of the* fimS* invertible element orientation by PCR on chromosomal DNA isolated from NU149 grown in LB as well as NU149 infected murine bladder and kidney homogenates spanning a five-day infection period. Two random different UPEC infected murine bladder and kidney homogenates were screened for each time point. Multiplex PCRs were set up with INV and FIMA primers to amplify Phase-ON-oriented DNA (ON, 450 bp product) [28], FIME and INV primers to amplify Phase-OFF-oriented DNA (OFF, 750 bp product) [41], and EcFtsZ 1 and 2 primers to amplify the ftsZ gene (302 bp product) [45]. Each multiplex was run at least three separate times. The lanes were loaded onto a 1.5* *% agarose gel as follows: lane 1, NU149 inoculum; lane 2, NU149 infected bladder mouse 1 day 0.33; lane 3, NU149 infected bladder mouse 2 day 0.33; lane 4, NU149 infected kidney mouse 1 day 0.33; lane 5, NU149 infected kidney mouse 2 day 0.33; lane 6, NU149 infected bladder mouse 1 day 1; lane 7, NU149 infected bladder mouse 2 day 1; lane 8, NU149 infected kidney mouse 1 day 1; lane 9, NU149 infected kidney mouse 2 day 1; lane 10, NU149 infected bladder mouse 1 day 3; lane 11, NU149 infected bladder mouse 2 day 3; lane 12, NU149 infected kidney mouse 1 day 3; lane 13, NU149 infected kidney mouse 2 day 3; lane 14, NU149 infected bladder mouse 1 day 5; lane 15, NU149 infected bladder mouse 2 day 5; lane 16, NU149 infected kidney mouse 1 day 5; lane 17, NU149 infected kidney mouse 2 day 5. For each lane, the intensities of the OFF and ON states were quantified using ImageQuant software (Molecular Dynamics) and corrected to the intensity of the ftsZ band. The corrected values for both states were standardized to the respective wild-type band (lane 1).

To confirm the orientation of the* fimS* element from NU149 cells infecting murine bladders and kidneys over a 120 hpi period, a PCR approach used previously [28, 45] was performed. Chromosomal DNA was isolated from several infected bladder and kidney homogenates for each time point, and a multiplex PCR was performed on each DNA sample. The inoculum had predominately Phase-ON oriented* fimS* (90.8% Phase-ON, 9.2% Phase-OFF, Figure 4). The 8 hpi samples for both infected bladder and kidney samples also showed the position of the* fimS* element predominantly in the Phase-ON orientation (89.6% Phase-ON and 88.1% Phase-ON, resp.). At 24 hpi the Phase-OFF orientation of the* fimS* element increased to 16.4% Phase-OFF in infected murine bladder homogenates and 16.8% to 26.2% Phase-OFF in infected murine kidney homogenates compared to the inoculum. By 72 hpi, the Phase-OFF position in the murine bladders averaged 19.1% Phase-OFF. However, the position of the* fimS* element in 72 hpi infected kidneys showed an average of 77.8% Phase-OFF orientation as compared to the inoculum lane. Finally, the 120 hpi results demonstrated a 77.2% Phase-ON and 22.8% Phase-OFF orientation for the* fimS* element within infected bladder homogenates compared to the inoculum. The greatest change in the positioning of the* fimS* element occurred in 120 hpi infected kidney homogenates where one sample had a 92.6% Phase-OFF and 7.4% Phase-ON orientation whereas the other kidney homogenate demonstrated 100% Phase-OFF and no Phase-ON oriented* fimS* element.

### 3.4. Production of Type 1 Pili Is Altered in a UPEC Strain Infecting Murine Bladders and Kidneys over a Five-Day Time Span

Both the* fim-lux* fusion and positioning of the fimS invertible element results changed in strain NU149 growing in murine bladders and kidneys over a five-day period. To determine if the level of type 1 pili expressed on the surface also changed in NU149 cells infecting murine bladders and kidneys, HA assays were done using guinea pig erythrocytes. A 512 HA titer was observed for the initial inoculum. The results showed that the HA titers from NU149 infected bladder homogenates fluctuated approximately twofold over the five-day infection (Table 2). However, the geometric means of the HA titers in NU149 infected kidney homogenates varied from 207.9 after 8 hpi to an HA titer of 0 after 120 hpi. Clearly, UPEC cells became more non-type 1 piliated over time in infected murine kidneys.

## 4. Discussion

The binding of type 1 piliated UPEC cells to epithelial cells lining the urinary tract is an important step in pathogenesis within the human or murine urinary tract. Environmental cues, such as pH and osmolality, within the urinary tract can regulate several key* fim* genes involved in UPEC type 1 pili expression. Even within the urinary tract, there are considerable changes in pH and osmolarity. For example, human bladder urine has a higher pH and lower osmolarity than kidney urine [29]. Murine urine has an even higher average osmolality [27]. In a previous study,* fim-lacZYA* fusions were used to examine the* in vitro* effects of pH and osmolality on the expression of* fimA*,* fimB*, and* fimE* gene. Growth of a UPEC strain in a low pH environment led to downregulation of* fimA*,* fimB*, and* fimE* gene involved in type 1 pili expression [28]. The use of* lacZYA* fusions in bacterial infected animal tissues is limited due to the need for bacterial cell lysis and the requirement for adding substrate when doing *β*-galactosidase assay. However, a* lux* fusion can be used without disruption of the bacterial cell membrane and loss of bacterial cell viability. Moreover, if an IVIS imaging system is available, the same mice could be assessed during many time points by merely anesthetizing the mice, which would hold an advantage over quantitative real-time polymerase chain reaction analysis. To assess* fim* gene regulation* in vivo*, we created a series of* fim-lux* reporter fusions on a single copy plasmid to assess how environmental cues affect transcription of* fimA*,* fimB*, and* fimE* in a UPEC strain infecting murine urinary tracts.

Initially, NU149 strains containing the* fim-lux* fusions were examined after* in vitro* growth in LB media with differences in pH to determine whether they matched the results using the* fim-lacZYA* fusions [28]. Most of the* in vitro* results with the* fim-lux* fusions were similar to the observations using the* fim-lacZYA* fusions. Both present and previous studies showed all of the* fim* genes had the lowest level of transcription in pH 5.5 LB medium. An exception was that transcription of* fimE* was the highest in pH 8.0 LB media in the present study, whereas the previous study showed optimal expression in pH 7.0 LB media [28].

Because most of the* in vitro fim-lux* fusion results correlated with the previous* fim-lacZYA* fusion study, we next assessed transcription from* fimA-*,* fimB-*, and* fimE-lux *fusion in a UPEC strain colonizing murine urinary tracts over a five-day postinoculation period. The* ftsZ-lux* control fusion worked well in UPEC infecting murine urinary tracts. Transcription of* ftsZ *did not significantly change in NU149 infecting bladders or kidneys, although* ftsZ* expression was lower on average in kidney homogenates compared to bladder homogenates. Thus,* ftsZ* transcription remained fairly stable over the five-day infection period in mice.

Our* in vivo fimA* transcription, invertible element PCR, and HA titer results were consistent with previous reports that showed the highest level of type 1 pili expression at 24 hpi in NU149 infected murine bladders. In one study, a cystitis isolate maintained the* fimS* Phase-ON orientation throughout the entire four-day period of bladder infection [37]. Other studies have shown that type 1 pili expression was most important for bacterial growth in the early stage (24 h after infection) of a UPEC infection in murine bladders and the* fimS* region remained mostly Phase-ON throughout the entire seven days bladder infection [34, 38, 40]. Previously,* E. coli* strain NU149 was also shown to maintain a consistently high degree of type 1 piliation in UPEC infected murine bladders after five days after inoculation [30, 32].

The* fimA* expression results from this study are in agreement with the concept that type 1 pili expression is needed in the initial stages of infection, but their expression is reduced once the UPEC cells attach and/or penetrate bladder epithelium [51, 52]. Human and murine bladder epithelial cells present an abundance of mannose moieties on their glycoproteins that may serve as receptors for type 1 pili [53], so continued production of type 1 pili would be advantageous for UPEC bladder colonization.

Like the* fimA* expression results from NU149 infected bladders, NU149 cells in infected murine kidneys also expressed the highest level of* fimA* transcription at 24 hpi and then displayed a significant drop in* fimA* transcription thereafter. No* fimA* transcription was detected in some of the 120 hpi infected kidney homogenates, suggesting that type 1 pili expression had been completely shut down in those NU149 infected kidneys. Our PCR results that examined the position of the* fimS* element as well as HA titer results coincided with the* fimA* transcription results. Other studies have also observed the loss of type 1 piliated UPEC cells over time in UPEC infected murine kidneys [30, 32, 54, 55].

Besides the differences in transcription of* fimA* gene observed in UPEC infection of murine urinary tracts, temporal regulation of both* fim* recombinase genes was also observed. Transcription of both* fimB* and* fimE* was the highest after 72 hpi in NU149 infected murine bladders. Nevertheless, relative fimB transcription went up 2.7-fold when comparing the 8 hpi to the 72 hpi time point, whereas relative* fimE* transcription increased 100-fold when comparing the 8 hpi to the 72 hpi time point, suggesting the temporal regulation of* fimB* and* fimE* in UPEC infecting murine bladders appears to favor* fimB* transcription in the early stage of the infection and switches at 72 hpi to one that more favored* fimE* transcription later. Thereafter,* fimB* expression fell by 0.3-fold after 120 hpi but remained at a high level. Conversely, transcription of* fimE* declined to barely detectable levels by 120 hpi in NU149 infected murine bladders.

Since FimB and FimE have roles in positioning of the* fimS* region that contains the* fimA* promoter [17, 18], the ratio of these proteins would have an indirect influence on* fimA* transcription by altering the orientation of the* fimS* region. More* fimB* and less* fimE *transcription would favor FimB-promoted recombination in the* fimS* region to the Phase-ON orientation, thus in turn, leading to higher type 1 pili expression. A drop in* fimA* transcription at day 3 could be the result of the ratio of FimB to FimE favoring FimE-promoted recombination. On the other hand, the decline in* fimA* transcription at day 5 after inoculation may be the result of environmental cues exerting a direct effect on the regulation of the* fimA* promoter or the expression of alternative site-specific recombinases, such as HbiF, IpuA, IpuB, IpbA, and LeuX [35, 56, 57].

Our infected bladder results were in agreement with the work of many others. These other studies have relied on other UPEC strains like NU14, UTI89, and CFT073 as well as different mouse strains like C3H/HeN, C57BL/6, and CBA/J [7, 30, 33, 35–38, 40, 55, 57, 58]. Although there was some degree of bacterial gene expression variability in these other studies, similar trends were observed in our study. Two recent RNA sequencing studies have shown that there is considerable gene expression variability between UPEC strains growing in the urinary tract [59, 60]. In one study, a difference in the percentage of the* fimS* element in the Phase-ON orientation for a cystitis strain compared to the pyelonephritis strain CFT073 within the urine of infected mice was observed. Initially, the median percentages of invertible elements in the Phase-ON orientation for F11 and CFT073 at the 4 h time point were 2% and 9.3%, respectively. At day 1, both strains displayed significant divergence in the orientation of the* fimS* region. Strain CFT073 had an increase in Phase-ON orientation to only 33.6%, while the Phase-ON orientation at the same time point for strain F11 increased to 84.5%. However, at day 2 and day 3 after inoculation, these two strains displayed a drop to ≤ 2% for strain CFT073 and 61.2% for F11 of the population in the Phase-ON orientation [37]. Our* fim* gene transcription results in NU149 infected murine bladders were also consistent with a recent study that showed* fimA* and* fimB* transcription declined over time in UPEC infected murine bladders [38].

In NU149 infected murine bladders, the temporal regulation of the* fim* genes favored* fimA* and* fimB* transcription in the early stage of the infections. However, in the murine kidneys, transcription of all three* fim* genes appeared to be repressed by 120 hpi. Transcription of* fimB* and* fimE* was the highest after 24 hpi in NU149 infected kidneys. Both* fimB* and f*imE* transcription were lower at the 72 hpi. By 120 hpi, relative* fimB* transcription dropped 12.4-fold compared to the 72 hpi time point, but* fimE* transcription had increased, suggesting the NU149 cells in the murine kidneys would have a relative ratio of* fimB* to* fimE* transcripts that favored* fimE* transcription and subsequently non-type 1 piliated cells by 120 hpi.

The question posed is why UPEC cell populations have their* fimS* element switch to the Phase-OFF orientation and lose their expression of type 1 pili over time in infected murine kidneys. Unlike bladder epithelial cells that have many mannose-containing receptors on their surface [61], kidneys display few of these receptors on their renal glycolipids [62], so expression of type 1 pili may be of little value to the bacteria in this environment. As UPEC strains ascend into the kidneys, the environmental niche they may encounter would have high osmolality conditions (800 mM NaCl equivalence) in pockets of the kidneys. Kidney urine has a lower pH than urine found in the bladders [29], so* fim* gene expression would be more repressed in this acidified/higher osmolality urine [28]. Another possibility is that the type 1 piliated UPEC cells are cleared more readily by macrophages that are more abundant in murine kidneys [63, 64]. We hypothesize that the lower number of type 1 piliated UPEC cells over time in murine kidneys is due to a combination of regulation of the* fim* genes and increased clearance of the type 1 piliated subpopulation.

Previously, it was shown that UPEC growing in urine affects expression of type 1 pili by changing the orientation of the* fimS* element to favor the Phase-OFF position [51, 65]. Furthermore, Greene et al. [65] demonstrated that human urine also had an effect on the function of type 1 pili by inhibiting the function of the type 1 pilus adhesion, FimH. We have also previously shown that human urine can repress transcription of both* fimA* and* fimB* [28]. Our current results with UPEC infected mice also show a reduction in* fimA* and* fimB* transcription that is coupled with more Phase-OFF oriented* fimS* and lower HA titers over time in murine kidneys bathed with murine urine.

To survive in a low pH/high osmotic stressed environment, the EnvZ/OmpR system would be needed by a UPEC strain. Previously, our laboratory showed* fimB* expression was derepressed in an* ompR* mutant UPEC strain in a low pH environment [28] and OmpR was critical for UPEC survival in the murine urinary tract [66]. Transcription of* ompR* in* E. coli* has been shown to be insensitive to fluctuations in pH [31, 67], but OmpR protein levels increased in UPEC grown in an acidic pH versus a neutral pH environment [31]. Compared to the wild-type parent, the decline in* fimB* transcription within UPEC infected kidneys could be the result of more OmpR protein being translated in this low pH/high osmolality environment. More OmpR protein would mean a greater opportunity to bind to the second* fimB* promoter and repress* fimB* expression [31] directly or maybe OmpR regulates other factors tied to regulating* fimB* and* fimE* [68]. Less* fimB *expression in UPEC infected kidneys could mean the ratio of FimB to FimE would change to favor FimE and a subsequent Phase-OFF orientation of the* fimS* region. With the* fimS* region being switched to a Phase-OFF orientation combined with direct regulation of* fimA* transcription, a loss of type 1 pili over time in UPEC infected kidneys would occur.

Why would non-type 1 piliated UPEC cells be advantageous in UPEC infected kidneys? Type 1 pili are highly immunogenic [69], so nonpiliated bacteria may be hidden from the host immune system that would otherwise opsonize the bacterial cells by antibodies binding to the type 1 pili on the UPEC surface. The murine kidneys are quite vascularized and macrophage-bacteria interactions would occur more often in murine kidneys as compared to bladders. Type 1 piliated bacteria are targeted directly by macrophages [63, 64], so non-type 1 piliated bacteria would hide behind their capsules and evade the murine innate defense. Thus, becoming non-type 1 piliated is an advantage for UPEC survival in the kidney, and the low pH/high osmolality environment encountered in the murine kidney would regulate the* fim* genes to favor a non-type 1 piliated phenotype.

Our* in vivo* results provided the evidence that different environmental niches within a UPEC infected murine urinary tract can regulate* fim* gene transcription, favoring expression of type 1 piliated cells in the murine bladders and non-type 1 piliated cells in the murine kidneys. Additional work is needed to investigate and clarify the molecular mechanisms that are shaped by the environment cues in a murine urinary tract that can influence type 1 pili expression.

## 5. Conclusion

Temporal regulation of the* fimA*,* fimB*, and* fimE* genes occurs in UPEC cells colonizing murine urinary tracts. Over time in murine kidneys, the ratio of* fimE* to* fimB* transcription switches to favor* fimE*, which results in the* fimS* element flipping to a more Phase-OFF orientation and the loss of type 1 pili on the surface of the UPEC cells within the murine kidneys.

## Figures and Tables

**Table 1:** Bacterial strains and plasmids used in this study.

Strains/plasmids	Description	Reference or source
*E. coli* strains		
NU149	Cystitis clinical isolate	[30]
DH5*α* MCR	General cloning strain	Gibco/BBL
Plasmids		
pAON-1	*fimA-lacZYA* locked Phase-ON on pUJ8	[28]
pP5-48	*fimB-lacZYA *on pUJ8	[28]
pMP5-2.17	*fimE-lacZYA *on pUJ8	[28]
pUJ8	*trp*′*-*′*lacZ phoA *Ap^r^	[42]
pHSS22	*ori*T Km^r^	[28]
pXen-5	Em^r^, promoterless *lux* operon	[43]
pPP2-6	pPR274 with multiple cloning site and Cm^r^	[28]
pWS141-2	*fimB* promoters on pHSS22	This study
pWS144-27	*fimB-lux* fusion on pHSS22	This study
pWS145-38	*fimB-lux* fusion on pPP2-6	This study
pHD-01	*fimA* promoter on pHSS22	This study
pHD-02	*fimA*-*lux* fusion on pHSS22	This study
pHD-03	*fimA*-*lux* fusion on pPP2-6	This study
pHD-04	*fimE* promoter on pHSS22	This study
pHD-05	*fimE*-*lux* fusion on pHSS22	This study
pHD-06	*fimE-lux* fusion on pPP2-6	This study
pHD-07	*ftsZ* promoter on pHSS22	This study
pHD-08	*ftsZ-lux* fusion on pHSS22	This study
pHD-09	*ftsZ-lux *fusion on pPP2-6	This study

**Table 2:** Measurement of hemagglutination (HA) titer for UPEC strain NU149 infecting murine bladders and kidneys over a five-day time period.

Organ	Hours after inoculation			
	8	24	72	120
Bladder	222.8^a^	119.4	114.4	104
Kidney	207.9	111.4	27.9	0

^a^HA titer represents the geometric mean from 10 different mouse organ preparations.
